# Atomic-Scale Interfacial Dynamics and Twin Formation in Cu/Al_2_Cu/Al Layered Composites During Cooling: Insights from Molecular Dynamics Simulations

**DOI:** 10.3390/nano15060437

**Published:** 2025-03-13

**Authors:** Shuang Li, Yunfeng Cui, Wenyan Wang, Jingpei Xie, Aiqin Wang, Feiyang Zhang, Zhiping Mao

**Affiliations:** 1 School of Materials Science and Engineering, Henan University of Science and Technology, Luoyang 471023, China; shuangbright_3230@126.com (S.L.);; 2 Digital Molding Engineering Research Center of Tungsten and Molybdenum Materials in Henan Province, Luoyang 471822, China; 3 Provincial and Ministerial Co-Construction of Collaborative Innovation Center for Non-ferrous Metal New Materials and Advanced Processing Technology, Henan University of Science and Technology, Luoyang 471023, China

**Keywords:** Cu/Al_2_Cu/Al system, molecular dynamics simulations, cooling

## Abstract

This study investigates the cooling process of the Cu/Al_2_Cu/Al system following high-temperature diffusion using molecular dynamics (MD) simulations based on an embedded atom method potential. The analysis focused on various characteristics to determine the structural and property changes within the Cu/Al_2_Cu/Al system during cooling. The findings reveal that only a small number of Cu atoms diffused along the Z-axis near the Cu/Al_2_Cu interface, while significant diffusion of Al atoms occurs in all directions at the Al/Al_2_Cu interface. Moreover, 673 K is identified as a crucial temperature for the crystal transformation of the Cu/Al_2_Cu/Al system during cooling. The Cu/Al_2_Cu interface exhibited migration behavior along the positive Z-axis. Additionally, the growth of Al_2_Cu towards the Al side resulted in a symmetrical lattice distribution along the Al/Al_2_Cu interface, leading to the formation of a twin crystal. In the AI layer, locally disordered atoms transform into vacancies under stress, accumulating as the temperature drops, thereby providing favorable conditions for dislocation initiation. Notably, cooling of the Al layer to 650 K led to the initial generation of 1/6<112> Shockley incomplete dislocations.

## 1. Introduction

Cu/Al laminated composites are widely used in power transmission and heat transfer engineering owing to their excellent electrical and thermal conductivity, lightweight nature, and cost effectiveness [1,2,3]. Common preparation methods include rolling compounds, explosive compounds, asynchronous rolling, and casting and rolling techniques [4,5,6]. Among them, casting and rolling a composite is the process of directly contacting a melted low-melting-point metal melt with a solid high-melting-point metal material, and rapidly solidifying under rolling pressure to form a composite interface. The high temperature of liquid metal ensures the mutual diffusion of copper and aluminum atoms in the interface layer, overcomes the disadvantages of a thick copper aluminum transition layer and low-interface-bonding strength, and achieves high-strength metallurgical bonding and wide-width production of copper and aluminum. The process is relatively simple, stable, and the equipment cost is low, which can adapt to large-scale continuous production [7,8].

Researchers generally believe that the solid–liquid composite of copper–aluminum composite materials results from a combination of fusion bonding and diffusion bonding [9]. When liquid aluminum comes into contact with solid copper, interfacial reactions occur at the contact surface, leading to the formation of intermetallic compounds from copper and aluminum atoms. Simultaneously, these atoms diffuse into each other, creating a diffusion layer that achieves metallurgical bonding at the composite interface. Concerning the composite mechanism during the casting and rolling process, Xie et al. [10] propose that the formation of the interface layer can be divided into four stages: first, copper and aluminum atoms diffuse into each other, forming supersaturated solid solutions near the interface defects; second, the supersaturated solid solutions on both sides of copper and aluminum nucleate and precipitate, resulting in the formation of the Al_4_Cu_9_ and Al_2_Cu phases; third, the Al_4_Cu_9_ and Al_2_Cu phases continue to grow and diffuse laterally along the interface, ultimately forming a continuous diffusion layer, while the AlCu phase nucleates and precipitates; finally, the AlCu phase grows laterally along the interface to form a separate reaction layer, with the three phases growing longitudinally together, leading to the development of a multi-layer structure at the interface. Liu Guoping [11,12] classified the formation of the interface layer during the casting and rolling process into six distinct stages, incorporating the formation process of the AlCu phase. Initially, α(Al) nucleation occurs, leading to the development of a cold zone on the surface of the copper substrate. Subsequently, high-temperature diffusion of copper and aluminum atoms transpires between these cold zones, causing the liquid aluminum matrix near the interface to begin supercooling, which results in the formation of solid aluminum. When the concentration of copper in aluminum surpasses a certain critical threshold, the ε_2_ phase begins to nucleate and grow, concurrently causing a decrease in the melting point of the aluminum matrix at the interface. The elevated heat subsequently remelts the quenching zone, creating an interface remelting zone. Following this, the interface undergoes a crystal inclusion reaction, where the ε_2_ phase gradually transforms into the Al_2_Cu phase. Additionally, the Al_4_Cu_9_ phase nucleates and grows between the copper and Al_2_Cu phases, while α(Al) dendrites or the Al_2_Cu phase nucleate within the remelted zone. Finally, as the remelting zone solidifies, intermetallic compounds develop, leading to the generation of new intermetallic compounds, specifically the AlCu phase, which results in an interface transition. The presence of intermetallic compounds in the interface layer is significant, as they constitute a hard, brittle phase with high electrical resistivity compared to the matrix of copper and aluminum. Consequently, their phase structure and orientation relationship with the matrix substantially restrict the plastic deformation capabilities of copper–aluminum composite materials, influencing various mechanical properties, including compression, tension, and the deep processing of copper–aluminum composite plates [13,14].

In recent years, despite rapid advancements in experimental equipment and imaging technology, the analysis of interface properties continues to encounter significant challenges. Concurrently, the recent progress in chip technology has facilitated theoretical calculations and simulations, compensating for the limitations of experimental research in this domain [15]. By employing atomic-scale simulations, researchers can extract more comprehensive insights into the thermodynamic and structural properties of solid–liquid interfaces. Molecular dynamics (MD) serves as a crucial numerical simulation method extensively utilized to investigate the interdiffusion processes between bimetallic materials. This approach enables the analysis of the diffusion behavior of metal atoms, providing an intuitive understanding of their diffusion and microscopic motion states. Currently, molecular dynamics is widely applied to the study of surface diffusion, bulk diffusion of single metals, and interdiffusion between dissimilar metals [16,17,18]. For example, Chu et al. [19] employed MD simulations to explore the mechanical properties of the Cu-Al interface layer at various diffusion temperatures. Their results indicated that both the equivalent phonon thermal conductivity and yield strength decreased with increasing temperature. Additionally, Chen Yao [20], a member of our research group, investigated the fundamental characteristics of micro-deformation within the Cu-Al interface system using MD simulations. Her research concentrated on the Cu/Al_2_Cu interface and the Al_2_Cu/Al interface, emphasizing their roles in inhibiting crack propagation and enhancing composite strength. Notably, during deformation, the crack within the Al_2_Cu layer did not propagate into the Cu layer, demonstrating that the Cu/Al_2_Cu interface significantly reinforces the composite material.

The casting–rolling process comprises two primary stages: cooling and rolling. During the cooling stage, molten aluminum undergoes rapid solidification under hydrostatic pressure, a process significantly influenced by the cooling rate, which critically determines the resultant phase formation [21]. Since nucleation begins within the supercooled melt, directly observing the nucleation process experimentally is challenging due to its opacity. Consequently, the evolution of the crystal structure during the cooling process of cast–rolled copper–aluminum layered composites can be investigated at the atomic scale using MD simulations. This work advanced the understanding of nanoscale interfacial dynamics in metallic composites by integrating molecular dynamics simulations with crystallographic analysis. The atomic-resolution insights into vacancy migration, dislocation nucleation, and twin formation provided a roadmap for optimizing nanolayered materials in applications such as flexible electronics, energy storage, and high-strength nanocomposites.

## 2. Models and Methods

The simulations in this study were conducted using the LAMMPS software package(LAMMPS 64-bit 15 June 2023), developed by Sandia National Laboratories [22]. The analytical embedded atomic method (EAM) potential proposed by Cai et al. was employed as the potential function in this study [23]. This atomic potential has been widely utilized in studies investigating atomic diffusion behavior in metals and intermetallic compounds [24]. The results are largely consistent with Miedema’s alloy theory and first-principle calculations.

**Figure 1:**
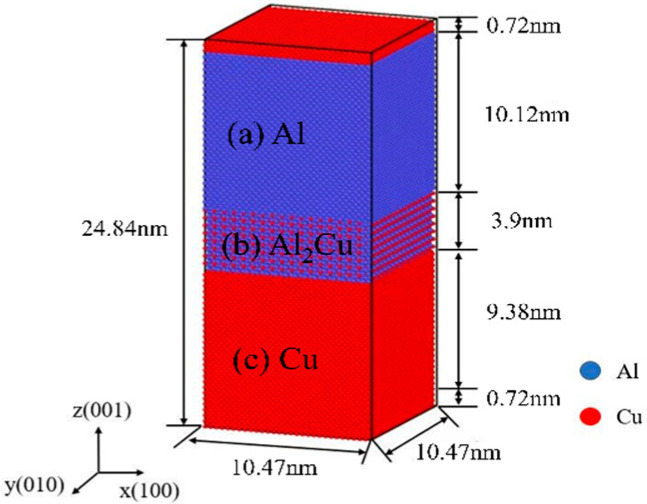
Cu/Al_2_Cu/Al multilayer structure: (**a**) Al; (**b**) Al_2_Cu; (**c**) Cu.

As shown in Figure 1, the initial model employs periodic boundary conditions along theX- and Y-axes, contraction-oriented boundary conditions in the positive Z-axis direction, and fixed boundaries in the negative Z-axis direction. The top and bottom layers are constrained in the Z-direction, with each layer consisting of two atomic layers. The model is predominantly composed of a Cu single-crystal structure, an Al_2_Cu single-crystal structure, and an Al single-crystal structure, with an ideal (001) plane serving as the contact plane. The specific atomic layer size is 10.47 nm in the X-direction and Y-direction, corresponding to 29 periodic Cu unit cells, 17 periodic Al_2_Cu unit cells, and 26 periodic Al unit cells. In the Z-direction, the specific atomic layer size is 24.84 nm, proportional to 26 periodic Cu unit cells, 8 periodic Al_2_Cu unit cells, and 25 periodic Al unit cells. The model consists of 196,264 atoms, which adhere to Newton’s laws of motion and are numerically integrated using the Verlet technique. The initial velocity of the atoms was determined using the Maxwell distribution, and the time step was set to 1 fs.

According to the fundamental theories of thermodynamic statistical physics, molecular dynamics simulations necessitate the establishment of a specific ensemble to create a relatively stable environment for the simulation object. Commonly utilized ensembles include microcanonical ensembles (NVE ensembles), canonical ensembles (NVT ensembles), and isothermal–isobaric ensembles (NPT ensembles) [25]. The NVE ensemble is defined by the independence of its internal atomic number (N), volume (V), and total energy (E) from external influences. It sustains the requisite energy through mutual conversion within the system while maintaining constant total energy and atomic number, thereby functioning as an independent statistical ensemble. In contrast, the NVT ensemble maintains a constant atomic number (N), volume (V), and temperature (T). This ensemble is kept in a state of thermal equilibrium, with temperature regulated through energy exchange with the external environment. The NPT ensemble holds the number of particles (N), pressure (P), and temperature (T) constant, with temperature control achieved by adjusting the velocities of the internal atoms. In MD simulations, controlling the temperature and pressure is essential for maintaining their constancy. Common methods for temperature control within ensembles include the velocity calibration method, Berendsen heat bath method, Gaussian heat bath method, and Nose–Hoover heat bath method [26]. Among these, the Nose–Hoover heat bath method is recognized for its capacity to more accurately reflect physical effects compared to other temperature control methods, making it a frequently employed technique in molecular dynamics simulations. This method modifies the Hamiltonian (H) of the simulation system by introducing a generalized coordinate that represents the heat bath, along with the corresponding generalized velocity of the heat bath. This coupling creates an isolated system within a micro-canonical ensemble, effectively linking the virtual constant-temperature heat source with the system under investigation [27]; the formula is as follows:

(1) $$H=\frac{1}{2}{\sum m_{i}(Sr_{i}’)}^{2}+V(r)+\frac{1}{2}Q\zeta^{2}+gKT\ln S$$

In this formula, *S* represents the generalized coordinate quantity of the heat bath, while *ζ* denotes the corresponding generalized velocity of the heat bath. The first term accounts for the total kinetic energy of the system, the second term corresponds to the potential energy of the system, the third term reflects the kinetic energy of the heat bath, and the fourth term signifies the potential energy of the heat bath. The motion equation of the system is expressed as follows:

(2) $$V_{i}=\frac{dr_{i}}{r}$$

(3) $$a_{i}=-\frac{\frac{dV}{dr}+m_{i}V_{i}Z}{m_{i}}$$

(4) $$\frac{dz}{dt}=\frac{\sum m_{i}V_{i}^{2}-gKT}{Q}$$

In this equation, z represents the friction factor, *Q* denotes the equivalent mass of the heat bath system, and g indicates the degrees of freedom of the system.

The simulation was conducted in the NPT ensemble, with temperature control via the Nose–Hoover thermostat, maintaining a simulated pressure of 1 atmosphere. Due to structural instability, the model initially contained substantial prestress and atomic potential energy. To prevent the destruction of the composite structure, it was crucial to relax each section of the model at 300 K for 50 ps before reassembling. The simulation process followed this sequence: initially, the system’s temperature was adjusted to 953 K; then, the system diffused for 200 ps at this temperature, and finally cooled down to room temperature (300 K) at a cooling rate of 1 × 10^11^ K s^−1^. This cooling rate is derived from previous relevant research [28,29,30]. During the metal crystallization process, the nucleation rate of crystals is influenced by the degree of supercooling. The short-range ordered arrangement of atoms, which is affected by energy fluctuations and structural variations near the crystallization temperature, plays a crucial role in nucleation [31]. As the degree of supercooling increases, it can enhance the nucleation of atoms within a specific range [32]. However, higher cooling rates adversely affect atomic diffusion, thereby inhibiting crystal growth. Given that both time and spatial scales are limited in molecular dynamics (MD) simulations, and considering that the nucleation of metal melts is a stochastic process, independent runs were necessary to achieve statistically significant results. In this study, each simulation was conducted 10 times.

## 3. Results and Discussion

### 3.1. Mean Square Displacement Analysis

Mean square displacement (MSD) is a statistical measure that quantifies the change in the position of a particle over a specified time interval. The diffusion of a material can be assessed by analyzing the variation in the mean square displacement with time. A material is considered to be diffusing if the mean square displacement demonstrates a linear increase with time; conversely, a slower or more gradual increase in mean square displacement indicates diminished or negligible diffusion. According to Einstein’s law of diffusion, the diffusion behavior of atoms is typically characterized by the relationship between mean square displacement and time. The diffusion coefficient, *D_sim_*, and the mean square displacement, *MSD*(*t*), are calculated using the following formula [26], where *N* represents the number of diffusing molecules in the system, *t* denotes time, *r_i_*(*t*) indicates the position of the particles at time *t*, and *r_i_*(0) signifies the position of the particles at time 0. The formula is as follows:

(5) $$D_{\mathrm{sim}}=\frac{1}{6N}\sum_{i=1}^{N}\underset{t\to \infty }{\lim}\frac{d}{dt}\langle {\left|r_{i}(t)-\left.r_{i}(0)\right|\right.}^{2}\rangle$$

(6) $$MSD(t)=\frac{1}{N}\langle \sum_{i=1}^{N}{\left|r_{i}(t)-r_{i}(0)\right|}^{2}\rangle =6D_{sim}t$$

**Figure 2:**
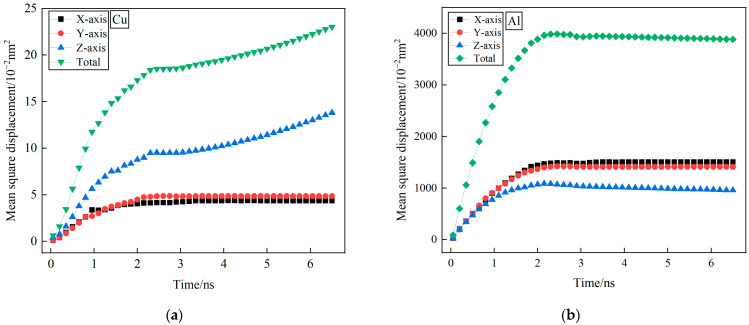
Variation curves of mean square displacement of Cu and Al atoms at the interface with time at the cooling rate of 1 × 10^11^ K s^−1^. (**a**) Cu atoms at the Cu/Al_2_Cu interface; (**b**) Al atoms at the Al/Al_2_Cu interface.

Figure 2 illustrates the progressive temperature reduction in the system, leading to an increasingly pronounced mean square displacement of Cu atoms as the simulation time advances. Specifically, Figure 2a,b highlights a linear increase in mean square displacement for Cu atoms along the Z-axis and Al atoms across the X- and Y-axes during the initial simulation period of 0 to 1 ns. This trend occurs before the cooling phase, where the system transitions from 953 K to 853 K. Notably, the slope of the mean azimuth shift remains relatively stable throughout this duration. These findings contribute valuable insights into the dynamics of atomic displacement within the studied system.

During the simulation period of 1 to 2.4 ns, there is a gradual decrease in the slope of the mean square displacement for both Cu and Al atoms. Notably, the slope of Al atoms tends towards zero. Subsequently, at the final simulation stage of 2.4 ns, corresponding to a system temperature of 713 K, the mean square displacement of Cu atoms in the Z direction exhibits a slight increase, while those in the X- and Y-directions remain relatively unchanged. Similarly, the mean square displacement of Al atoms in all directions displays minimal changes, aligning with the overall trend in total mean square displacement.

These findings suggest that during the cooling process, only a limited number of Cu atoms near the interface along the Z-axis undergo displacement, while extensive diffusion of Cu atoms in all directions at the Cu/Al_2_Cu interface is notably absent [26]. Cu atoms exhibit negligible average directional shifts, resulting in extremely low diffusive velocities and high diffusive resistances. In contrast, at the Al/Al_2_Cu interface, numerous Al atoms diffuse extensively in all directions, with similar changes observed in diffusion rates. This trend is consistent with Liu Guoping’s experimental results [11]. In the initial stage of cooling, the liquid aluminum atoms exist in a high-temperature disordered state. Upon contact with the relatively low-temperature copper matrix, the interface chilling effect leads to the formation of a thin, fine-grained region at the solid–liquid interface. Consequently, the interface temperature drops instantaneously from its peak value, resulting in the nucleation of α(Al) around 923 K, which subsequently grows to a certain extent. Interdiffusion occurs between copper and aluminum, accompanied by a phase change process near the temperature of 853 K. When examined in conjunction with the Al-Cu phase diagram, this phenomenon can be identified as the Al-Cu eutectic reaction zone, corresponding to the formation temperature range of the Al_2_Cu phase. This finding aligns with the research results of Pintore et al. [33]. As the cooling process continues, the interface temperature decreases further, leading to the nucleation and growth of the Al_2_Cu phase at the interface, while the Cu concentration continues to increase. However, due to the significantly lower solid solubility of copper in aluminum compared to that of aluminum in copper, the diffusion of Cu atoms is limited. It is therefore speculated that Al atoms near the interface will diffuse throughout the entire Al layer and Al_2_Cu layer, resulting in a uniform distribution.

Based on Einstein’s diffusion equation, the self-diffusion coefficients of Cu and Al atoms were determined by analyzing the time evolution of their mean squared displacement (MSD) at 900 K. The calculated diffusion coefficients along the X-, Y-, and Z-axes are summarized in Table 1. Our simulation results show good quantitative agreement with the values in the literature, exhibiting minor numerical discrepancies. The simulation utilized an ultra-fast cooling rate of 0.1 K ps^−1^, driving the system into a non-equilibrium glassy state. Here, transient kinetics dominate over equilibrium behavior: Early-stage (<6 ns) Cu atoms, benefiting from a smaller mass and higher mobility, preferentially form local order along the Z-direction ([110] crystallographic direction). Al atoms, exhibiting slower diffusion kinetics, retain significant disorder, leading to suppressed Z-direction movement. This transient anisotropy is captured within our short simulation window (≤6 ns), which may not fully equilibrate Cu’s Z-direction diffusion (requiring >100 ns for convergence). Consequently, discrepancies with research equilibrium data arise from both computational time limitations and potential inaccuracies in interatomic potential models (e.g., EAM potentials for Cu-Al interactions).

Notably, Cu and Al atoms exhibit nearly identical self-diffusion coefficients along the X- and Y-axes. Notably, Cu demonstrates a slightly higher coefficient in the Z-direction relative to the X/Y-axes, whereas Al displays a significantly lower coefficient in the same orientation. The anisotropy observed arises from fundamental differences in the crystallographic properties and dynamic behavior of Cu and Al. Both Cu and Al crystallize in face-centered cubic (FCC) structures but exhibit distinct lattice parameters (Cu:a = 3.614 Å; Al:a = 4.086 Å). At 900 K, enhanced thermal vibrations facilitate vacancy-mediated diffusion. The tighter Cu lattice (smaller atomic spacing) results in lower vacancy formation energies, promoting faster diffusion rates. Moreover, Cu’s smaller atomic radius (r_Cu = 1.28 Å vs. r_Al = 1.43 Å) enables easier Z-direction transitions through adjacent vacancy sites, whereas Al’s larger size and higher layer packing density in the FCC structure restrict Z-axis diffusion. These findings underscore the critical role of crystal anisotropy and non-equilibrium dynamics in dictating diffusion behavior during high-temperature processing.

### 3.2. Velocity Contour Analysis

**Figure 3:**
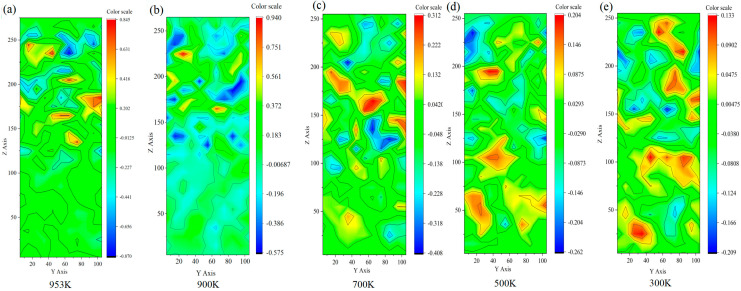
Velocity contour of the interface of the whole system from casting and rolling temperature cooling to room temperature. (**a**) 953 K; (**b**) 900 K; (**c**) 700 K; (**d**) 500 K; (**e**) 300 K.

As the system is cooled from casting temperature to room temperature, Figure 3 presents the velocity distribution of the atoms along the X-axis, illustrating their diffusion within the model. From Figure 3a–e, it is evident that the diffusion rate of copper atoms in the copper side region initially increases as the temperature decreases from 953 K to 900 K. However, as the temperature continues to drop, a gradual decline in the copper atom diffusion rate is observed. During the cooling process from 700 K to 300 K, a noticeable diffusion of Cu atoms occurs near the Cu/Al_2_Cu interface, aligning well with experimental observations. At 953 K, both the Al layer and the Al_2_Cu layer show six maximum and two minimum values in the velocity cloud image. This indicates the presence of lattice defects at these positions. The presence of either vacancy or gap sites, which could facilitate the diffusion of Al atoms, is highlighted by the maximum value in the velocity cloud image [36].

In the velocity cloud map, the lowest point corresponds to a lattice dislocation that causes a sudden change in the atomic arrangement. The presence of dislocation lines, planes, and bodies restricts the diffusion of atoms within the crystal [20].

Throughout the cooling process from the casting and rolling temperatures to room temperature, the velocity values of both the Al layer and Al_2_Cu layer experience multiple sudden changes. At each temperature, atoms at identical positions exhibit varying velocities due to the system-wide temperature decrease, leading to a continuous deceleration in diffusion rate and reduction in the thermal energy of Al and Cu atoms [37]. This reduction in thermal energy weakens the ability of Cu and Al atoms to overcome lattice obstacles and dislocations, limiting their atomic movement range and diffusion distance. This explains the changes in atomic velocity observed at various locations in the system. Additionally, a temperature decrease affects the diffusion behavior of Cu and Al atoms. At high temperatures, diffusion is driven by the free movement of atoms, whereas at low temperatures, the presence of lattice defects influences diffusion mechanisms and behaviors, impacting the diffusion rate. Due to the complex and non-uniform nature of the system, particle interaction between particles leads to an uneven spatial distribution of temperature. Elevated temperatures cause disordering of aluminum atoms, and the subsequent return to room temperature results in the formation of voids. These voids impede heat transfer, leading to uneven heating, which further affects atomic diffusion rates [38].

### 3.3. Distribution of Cu Atoms and Change in Model Volume During Cooling

**Figure 4:**
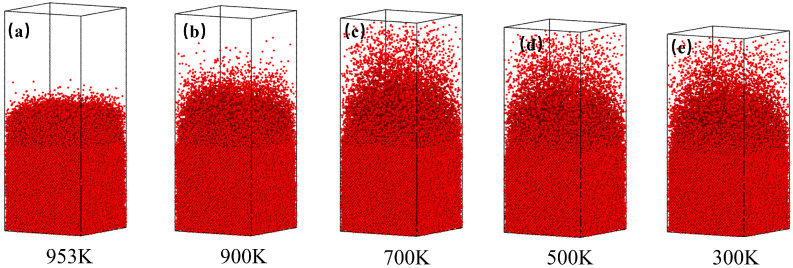
Cu atom distribution in the cooling system: (**a**) 953 K; (**b**) 900 K; (**c**) 700 K; (**d**) 500 K; (**e**) 300 K.

Figure 4a–e clearly shows that Cu atoms continuously diffuse toward the Al side during cooling, with their mixing degree significantly influenced by temperature. Within the high-temperature range (953–900 K), Cu atoms exhibit sparse and localized clustering (red points), indicating rapid diffusion kinetics despite incomplete mixing. As temperature decreases further, the diffusion range of Cu atoms expands progressively, accompanied by a gradual blurring of the Cu-Al interface, reflecting temperature-dependent deceleration of diffusion. Ultimately, at 300 K, Cu atoms achieve uniform distribution throughout the Al_2_Cu/Al composite system.

This reduction in diffusion can be attributed to two primary factors. Firstly, as the temperature decreases, the diffusion coefficient of Cu atoms diminishes, lowering the probability that Cu atoms will acquire sufficient energy to overcome potential barriers, thus slowing their diffusion rate. Secondly, the thickness of the newly solidified layer increases as the temperature decreases, hindering further mutual diffusion of Cu and Al atoms.

**Figure 5:**
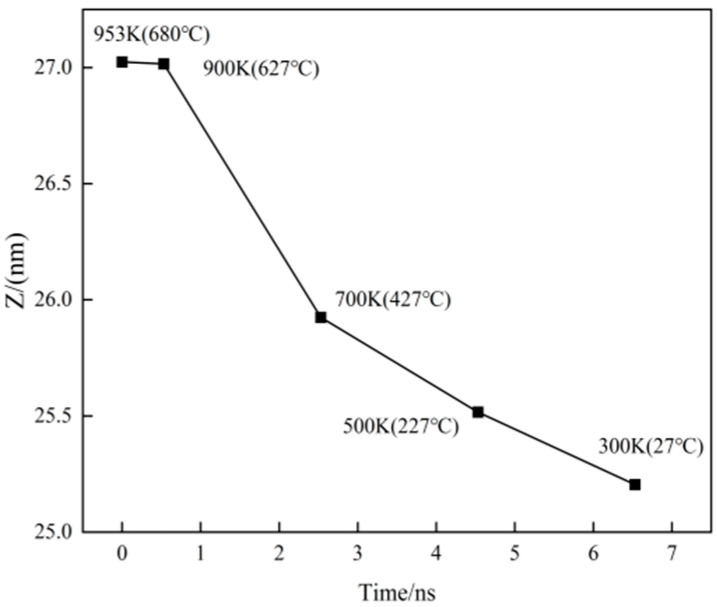
The variation curve of model height with time during cooling.

The model uses periodic boundary conditions along the X- and Y-axes, while the positive Z-axis is governed by contraction-oriented boundary conditions. Consequently, changes in the height of the Z-axis reflect variations in the volume of the model during the cooling stage. As shown in Figure 5, the model’s volume gradually decreases as the temperature is reduced from 953 K to 900 K. Within this temperature range, Cu and Al atoms exhibit high activity, engaging in heat exchange and mutual diffusion. The extent of their mixing will influence the distribution of the resulting phase upon cooling to room temperature. At 900 K, a rapid contraction occurs, which then transitions into a gradual and linear contraction at a specific temperature, potentially indicating the crystal transition temperature characterized by significant alterations in the atomic arrangement [30].

### 3.4. The Key Temperature of Crystal Transformation During Cooling

**Figure 6:**
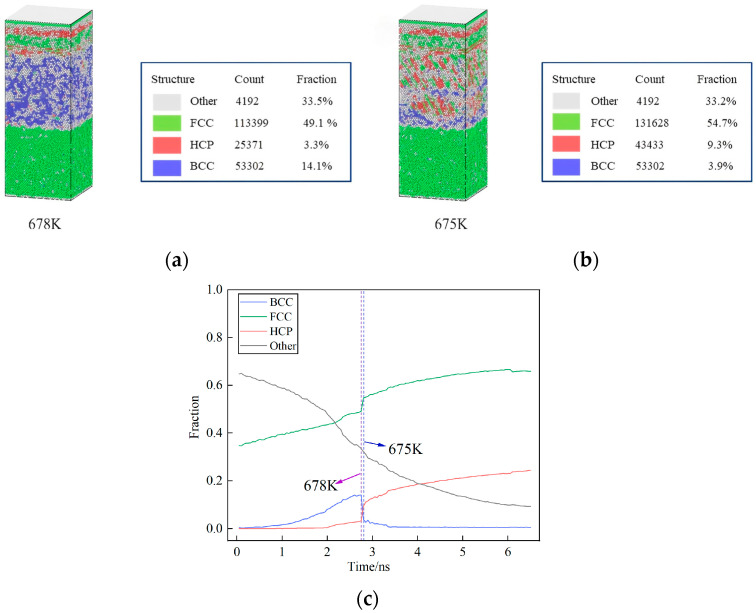
Common neighbor analysis diagram of the model at 678 K and 673 K and crystal proportion curve with time. (**a**) 678 K; (**b**) 673 K; (**c**) Crystal proportion curve with time.

Common Neighbor Analysis (CNA), originally proposed by Honeycutt and coworkers [39], is a method to characterize atomic stacking structures by quantifying the bonding relationships between atoms and their shared neighbors. In this study, we employ the adaptive common neighbor analysis (ACNA) implemented in the Open Visualization Tool (OVITO). Unlike conventional CNA approaches, ACNA dynamically adjusts the cutoff radius r_cut_ based on real-time neighbor position information, enabling enhanced accuracy for multi-phase system analysis [40]. The cooling rate employed in this study was set at 1 × 10^11^ K s^−1^. As illustrated in Figure 6c, there is a significant change in the proportions of crystal structures in the model between the simulation times of 2.75 ns and 2.8 ns. The face-centered cubic (FCC) and hexagonal close-packed (HCP) exhibit a notable increase, while the body-centered cubic (BCC) structure experiences a significant decrease. This shift occurs within a temperature range of 678 K to 673 K. Figure 6a,b demonstrates the changes in the distribution of crystal types within the model as the temperature decreases from 678 K to 673 K. At 678 K, the model consisted of a 49.1% FCC structure, a 3.3% HCP structure, and a 14.1% BCC structure. These proportions change to 54.7%, 9.3%, and 3.9%, respectively.

The process and mechanism of crystal transformation are complex and influenced by factors such as cooling temperature and rate, though this study does not explore the effects of varying cooling rates. It is speculated that the transformation observed may result from the formation of amorphous structures by Cu and Al at high temperatures. As cooling continues, various solid solutions with differing lattice parameters and atomic spacing begin to form. Due to Cu’s high melting point, Al atoms gradually diffuse towards the Cu side through the Al_2_Cu/Cu boundary, though only to a shallow extent. Consequently, as the temperature decreases, the Cu region in the model assumes an FCC metal structure, while other areas adopt a metastable crystalline FCC metal structure [18].

As cooling progresses further, the metastable FCC metal structure undergoes a phase transition, resulting in its transformation into a BCC structure. In proximity to this transition point, specific atoms within the BCC crystals gradually deviate from their original positions, leading to the occurrence of prismatic distortion. This distortion gradually spreads from a single point to encompass the entire crystal. Once the distortion reaches a certain degree, the atoms within the crystal reorganize themselves, adopting a different FCC or HCP structure that exhibits higher energy stability. Chen Xiaohua et al. [30] cooled the aluminum melt to 600 K using various cooling rates (2 × 10^11^ K s^−1^, 2 × 10^12^ K s^−1^ and 2 × 10^13^ K s^−1^), followed by isothermal treatment. The study revealed that upon the complete solidification of aluminum, two predominant types of atomic structures were observed: FCC and HCP. Notably, at a cooling rate of 2 × 10^11^ K s^−1^, two clusters emerged in the aluminum melt within a timeframe of 10 picoseconds. These clusters continued to grow by 20 ps, eventually developing into two larger clusters by 100 ps. The atoms within these clusters are predominantly an FCC structure, with some HCP structure present in the form of stacking faults. Over time, the two nuclei gradually grow and consume each other. Between 150 ps and 500 ps, the liquid phase in the system is depleted, and as some HCP structures are ablated and replaced by the FCC structure, the grains begin to grow.

### 3.5. The Formation and Evolution of Dislocation Lines

**Figure 7:**
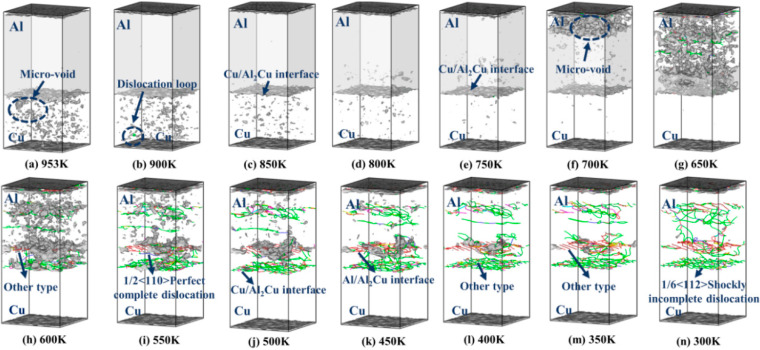
The formation and evolution of Cu/Al_2_Cu/Al dislocation line from casting temperature cooling to room temperature. (**a**) 953 K; (**b**) 900 K; (**c**) 850 K; (**d**) 800 K; (**e**) 750 K; (**f**) 700 K; (**g**) 650 K; (**h**) 600 K; (**i**) 550 K; (**j**) 500 K; (**k**) 450 K; (**l**) 400 K; (**m**) 350 K; (**n**) 300 K.

Figure 7 illustrates the formation and progression of dislocation lines during the cooling process of the entire system from the casting temperature to room temperature. The red dislocation line represents Other dislocation, the blue dislocation line indicates Perfect dislocation, the green dislocation line corresponds to Shockley dislocation, the purple dislocation line signifies Stair-rod dislocation, and the yellow dislocation line denotes Hirth dislocation. At a temperature of 953 K, the Al-Al bonds within the Al lattice undergo significant breakage due to thermal influences. Consequently, the ordered structure of the Al layer is disrupted, leading to a relatively disordered state, as illustrated in Figure 7a. The atoms within the Al layer undergo a transition from an ordered arrangement to a disordered configuration as a result of the temperature influence. As the temperature gradually drops from 953 K to 700 K, the model undergoes continuous volume contraction, with strain along the Z-axis increasing. This increased strain results in the formation of vacancies at the top of the Al layer, as depicted in Figure 7f.

With further cooling to 650 K, the accumulation of vacancies provides favorable conditions for the initiation of dislocations [16]. Under external loading, the first type of dislocation to appear within the Al layer is the 1/6<112> Shockley incomplete dislocation. This specific dislocation is illustrated in Figure 7g. Upon reducing the temperature to 600 K, different types of dislocations emerge at the Al/Al_2_Cu interface in the initial model. Notably, both the Al and Al_2_Cu layers still exhibit a significant number of vacancies, as showcased in Figure 7h.

As the temperature decreases to 300 K, the number of vacancies gradually diminishes until they are no longer present. Within the Al layer and at the Cu/Al_2_Cu interface of the initial model, 1/2<110> Perfect dislocations are generated. Additionally, it was observed that at the Cu/Al_2_Cu interface, the predominant distribution of dislocation lines consists of 1/6<112> Shockley incomplete dislocations, intermixed with a small quantity of shorter 1/2<110> Perfect dislocations. At the Al/Al_2_Cu interface, the main distribution of dislocation lines comprises other types of dislocations, along with a minor presence of shorter 1/2<110> Perfect dislocations and 1/6<112> Shockley incomplete dislocations. An interface is formed within the Al layer of the initial model, where the distribution of dislocation lines includes 1/6<112> Shockley incomplete dislocations and various other types of dislocations, as illustrated in Figure 7n.

The presence of 1/2<110> Perfect dislocations and 1/6<112> Shockley incomplete dislocations significantly influences the interface stability, mechanical properties, and deformation mechanisms of cast–rolled copper–aluminum layered composite materials. A 1/2<110> Perfect dislocation indicates that the movement of the dislocation line within the crystal structure is accompanied by the complete slippage of the atomic plane. This phenomenon effectively facilitates dislocation movement, promotes the migration and reconstruction of grain boundaries, and enhances the combined strength of the interface, thereby maintaining relative stability during the deformation process. It reduces stress concentration at the interface, minimizes the occurrence of cracks, and improves the material’s plasticity. In contrast, the movement of the 1/6<112> Shockley incomplete dislocation line does not involve complete atomic layer slip, which can lead to local distortion and stress concentration. While this type of dislocation may locally enhance the material’s strength, an excessively high dislocation density can diminish the bonding strength of the interface and result in brittle failure, making the material more susceptible to fracture under external forces. According to the results presented in Figure 6, it is evident that Cu/Al_2_Cu exhibits a greater number of dislocation lines, specifically those represented by Shockley dislocation lines, at room temperature, while fewer dislocation lines are observed at the Al/Al_2_Cu interface. This suggests that, during the deformation process, local delamination at the interface initiates the generation of dislocations in the metal intercompound Al_2_Cu layer. As strain increases, the crack front detaches from the Cu layer and propagates into it until the Cu layer ultimately fails. Following this failure, the crack extends into the Al layer, leading to a complete fracture of the composite plate.

**Figure 8:**
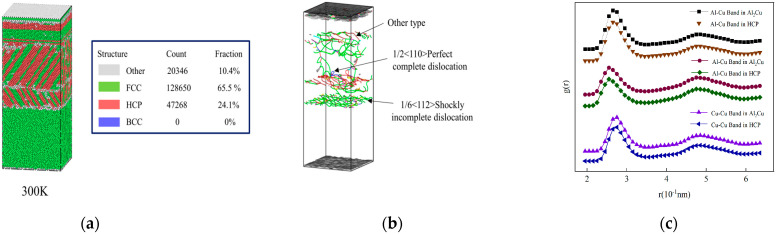
Model analysis after cooling to room temperature: (**a**) Common neighbor analysis diagram of the model at 300 K; (**b**) Dislocation analysis of the model at 300 K; (**c**) The radial distribution function of the HCP structure.

**Figure 9:**
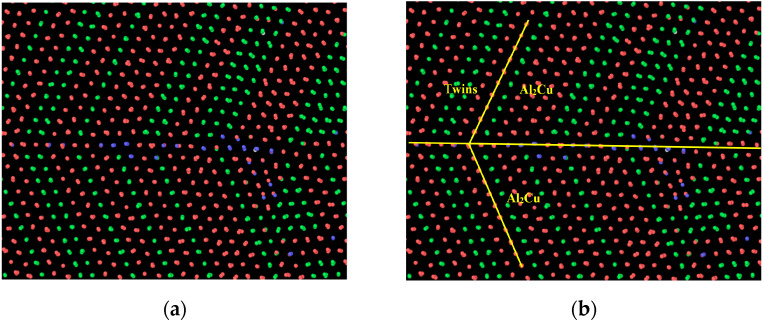
Polyhedron template matching analysis diagram and twin calibration at 300 K in the section direction of model (110). (**a**) Lattice structure mapping; (**b**) Twin formation and phase segregation analysis.

Figure 8a–c presents the results of common neighbor analysis, dislocation analysis, and radial distribution function analysis, respectively, for the HCP structure generated after cooling to room temperature. The radial distribution function of the resulting HCP structure closely aligns with that of the original Al_2_Cu configuration, thus confirming its identity as Al_2_Cu [41]. An examination of Figure 8a,b reveals a symmetric atomic distribution along the Al/Al_2_Cu interface at room temperature (300 K), suggesting the formation of twins within Al_2_Cu. Notably, the exclusive formation of Al_2_Cu on the Al side, rather than on the Cu side, indicates a preferential growth towards the Al side during cooling. However, it is important to note that conclusions should not rely solely on atomic symmetry; a thorough assessment of twin relationships necessitates a detailed analysis of the lattice structure and atomic symmetry. In Figure 9a, the Polyhedral Template Matching (PTM) analysis illustrates a cylindrical representation for atoms, facilitating the visualization of crystal orientation [42]. Importantly, the lattice distribution of Al_2_Cu along the Al/Al_2_Cu interface exhibits symmetry when viewed from the direction of the (110) crystal face, which is indicative of twin crystal formation. Dislocation analysis (DXA) reveals the presence of numerous Shockley incomplete dislocations, characterized by a Burgers vector of 1/6<112>, at the Al/Al_2_Cu interface. Additionally, a significant number of other dislocation types are observed at the Cu/Al_2_Cu interface.

### 3.6. Atomic Number Density Analysis of Cu and Al During Cooling

The analysis of number density distribution is an essential parameter for investigating the perpendicular direction’s ordering within an interface. In this study, the Cu/Al_2_Cu/Al system is discretized into numerous thin layers with a length of 0.01 nm along the Z-axis. Subsequently, the count of Cu atoms, Al atoms, and the total number of particles within each thin layer is determined. By dividing the volume of each thin layer, a detailed distribution of number density along the solid–liquid interface is ultimately obtained.

**Figure 10:**
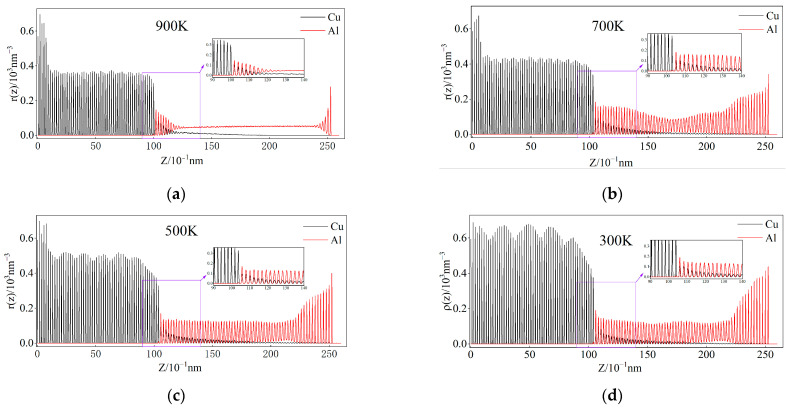
Distribution of Cu and Al atomic number densities along the normal direction of the interface at different temperatures during cooling.(**a**) 900 K; (**b**) 700 K; (**c**) 500 K; (**d**) 300 K.

Figure 10 provides an illustration of the nucleation and growth processes in ordered phases, which can be understood by examining the variations in atomic number density of Cu and Al during the cooling process. The presence of density peaks corresponds to atomic planes, allowing for an analysis of Cu and Al atom diffusion during cooling [23]. During the cooling process, overlapping density peaks of Cu and Al atoms appear within the diffusion interface region (approximately 9–14 nm), suggesting the merging of Cu and Al atomic planes post-solidification. By comparing the enlarged density distribution diagrams at 900 K and 700 K, we observe that, at 700 K, the peak density of Cu atoms increases in atomic planes several layers away from the Cu side, approximately 11 nm, compared to 900 K. This suggests the diffusion of Cu atoms towards the Al side following interface solidification.

Additionally, the number density distribution of Al atoms depicts the cooling process of liquid Al. At the cooling interface and at 900 K, a peak in Al atomic number density is observed on atomic planes near the copper side. As the temperature decreases, these atomic planes gradually shift towards the right and stabilize at 500 K, indicating interface migration behavior from copper to the Al side [43]. In the enlarged 300 K number density distribution figure, it is evident that the peak number density of Al atoms remains constant. The atomic number density of Al atoms is higher on atomic planes near the Cu side of the solidified interface layer, while the atomic number density of Cu atoms is lower. On one atomic plane, a ratio of ρ(z)_Cu_:ρ(z)_Al_ = 1/2 is observed. Considering diffusion and phase transition kinetics, it can be inferred that the forming phase at the interface is Al_2_Cu. It is important to note that technical terms like “phase transition kinetics” and “diffusion kinetics” should be appropriately defined. Moreover, a ratio of ρ(z)_Cu_:ρ(z)_Al_ > 1/2 indicates the coexistence of Al_2_Cu and Cu atoms on other atomic planes [43].

## 4. Conclusion

During the cooling process, a small number of Cu atoms near the Cu/Al_2_Cu interface diffuse along the Z-axis, while significant diffusion of Al atoms occurs in all directions at the Al/Al_2_Cu interface. This diffusion leads to a migration of the Cu/Al_2_Cu interface from the Cu side towards the Al side. Certain regions within the Al and Al_2_Cu layers contain vacant or gap sites, which facilitate the movement and diffusion of Al atoms by providing easier pathways. In contrast, other regions exhibit lattice dislocations, causing rearrangements in atomic positions and impeding diffusion within the crystal due to the presence of dislocation lines, planes, and bodies.

A critical temperature of 673 K plays an essential role in the crystal transformation process, resulting in a more stable crystalline structure. Additionally, the growth of Al_2_Cu crystals towards the Al side leads to a symmetrical lattice distribution along the Al/Al_2_Cu interface, ultimately resulting in the formation of a twin crystal. Throughout the cooling process, locally disordered atoms in the Al layer transform into vacancies under the influence of stress, with these vacancies accumulating as the temperature decreases. This accumulation creates favorable conditions for dislocation initiation, and, at a temperature as low as 650 K, 1/6<112> Shockley incomplete dislocations are first observed within the Al layer.

## Figures and Tables

**Table 1:** The diffusion coefficients of Cu and Al atoms were determined at a cooling temperature of 900 K under rapid-cooling conditions.

	Diffusion Coefficient of Cu (m^2^ s^−1^)			Diffusion Coefficient of Al (m^2^ s^−1^)		
Diffusion Coefficients	This Work (900 K)	R^2^ Value	Others’ Data	This Work (900 K)	R^2^ Value	Others’ Data
D	3.16 × 10^−9^	0.996	4.83 × 10^−10^ (893 K) [34] 4.08 × 10^−10^ (813 K) [35] 1.32 × 10^−10^ (733 K) [36]	6.81 × 10^−9^	0.997	6.24 × 10^−9^ (893 K) [34] 2.39 × 10^−9^ (813 K) [26] 8.18 × 10^−15^ (733 K) [35]
D_X_	8.52 × 10^−10^	0.995	--	2.35 × 10^−9^	0.995	--
D_Y_	7.56 × 10^−10^	0.995	--	2.43 × 10^−9^	0.995	--
D_Z_	1.55 × 10^−9^	0.997	--	2.03 × 10^−9^	0.996	--

## Data Availability

The original contributions presented in this study are included in this article. Further inquiries can be directed to the corresponding author.
